# P-1881. Don't Know Much About Geography? Decision Support for Frontline Clinicians Evaluating Patients with Suspected High Consequence Infectious Diseases

**DOI:** 10.1093/ofid/ofae631.2042

**Published:** 2025-01-29

**Authors:** Jacob E Lazarus, Lindsay Germaine, Michelle S Jerry, Chloe V Green, Erica S Shenoy

**Affiliations:** Massachusetts General Hospital, Boston, Massachusetts; Mass General Brigham, Somerville, Massachusetts; Massachusetts General Hospital, Boston, Massachusetts; Massachusetts General Hospital, Boston, Massachusetts; Mass General Brigham, Somerville, Massachusetts

## Abstract

**Background:**

High Consequence Infectious Diseases (HCIDs) are acute infectious diseases characterized by high case-fatality rates, few (if any) rapidly accessible treatments, and person-to-person spread in the absence of appropriate transmission-based precautions. Rapid identification and isolation of suspected cases is critical, however, diagnosis is challenging because HCIDs can manifest with nonspecific symptoms and specialized knowledge about the geography of current outbreaks, as well as HCID-specific exposure risk factors and incubation periods, are not uniformly known to healthcare personnel (HCP) performing patient evaluations. Clinical Decision Support Systems (CDSS) embedded within the electronic health record (EHR) may facilitate assessment.

**Figure 1:**
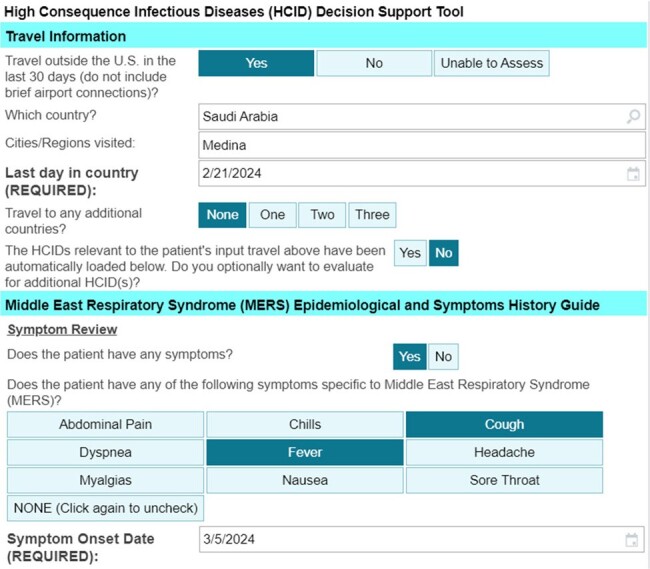
EvalHCID Clinical Decision Support System Demonstration From top to bottom, EvalHCID automatically imports travel information from the routine travel screening conducted during initial patient triage. Additional travel can be manually entered, with an itinerary of up to 4 countries. By cross-referencing a system-wide database of circulating HCIDs, EvalHCID automatically loads an interface to evaluate HCIDs relevant to the patient’s travel history. By subtracting symptom onset date from the last day in country, symptom development outside the relevant incubation period can be identified.

**Methods:**

We describe the design and development of an EHR-embedded CDSS tool, linked to a travel and symptom screening, and referencing a regularly updated worldwide HCID Watch List, to assist HCP in the evaluation of a returning traveler with infectious symptoms. Performance and user experience of the tool are described.

**Figure 2:**
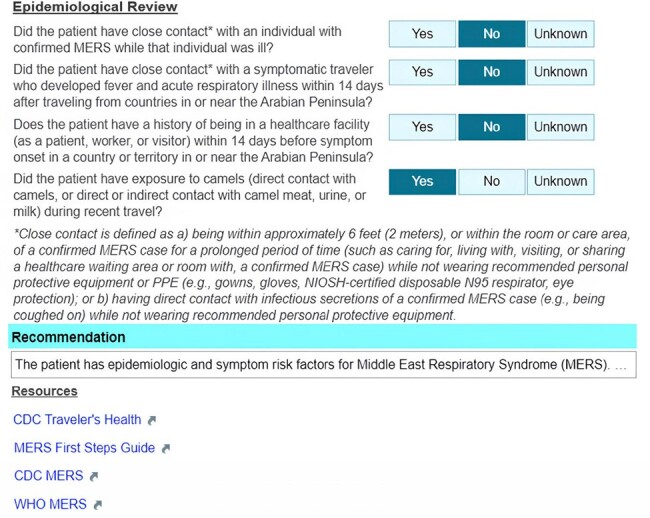
EvalHCID Clinical Decision Support System Demonstration From top to bottom, EvalHCID facilitates collection of epidemiological history that would place a traveler at high risk for the development of an HCID (in this case, the Middle East Respiratory Syndrome). Based on the data entered into EvalHCID, a recommendation regarding risk stratification and next steps is given. Relevant resources are also provided.

**Results:**

The Evaluate for High Consequence Infectious Disease (EvalHCID) CDSS facilitates identification of patients with travel, symptom, and epidemiological risk factors warranting Person Under Investigation (PUI) status for viral hemorrhagic fevers (including Lassa Fever, Crimean-Congo Hemorrhagic Fever, Marburg Virus Disease, and Ebola Virus Disease) as well as Novel Influenza and the Middle East Respiratory Syndrome (Figures 1 and 2). Using integrated, adaptable logic concordant with public health guidance, EvalHCID can be deployed to rapidly identify patients who meet PUI criteria, while allowing patients who do not meet PUI criteria to proceed with an otherwise appropriate clinical evaluation (Figure 3).

**Figure 3:**
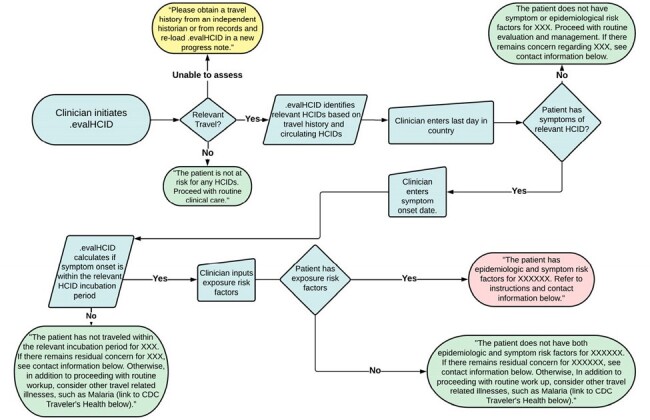
EvalHCID Clinical Decision Support System Logic By incorporating travel history, the dates of travel and symptom onset, relevant symptoms, and epidemiological risk factors, EvalHCID assists frontline healthcare personnel in risk-stratifying returning travelers for HCIDs.

**Conclusion:**

With highly specialized and accessible HCID expertise embedded within the EHR, HCP identification of patients at high risk for HCIDs is augmented, enabling rapid initiation of appropriate isolation when indicated for PUIs. At the same time, through speeding the evaluation of low-risk patients, care can be improved by allowing diagnosis of more likely clinical conditions.

**Disclosures:**

All Authors: No reported disclosures

